# The Late King and the Hospitals

**Published:** 1911-03-04

**Authors:** 


### The Late King and the Hospitals.

**Note.—We shall be glad to receive information regarding further memorials in which alterations to existing institutions or the establishment of new hospitals are contemplated, in order to amplify this provisional list. Details about the estimates of cost and the nature of the schemes adopted will be acceptable.—Editor, "The Hospital."**

**Dundee.**—An extension of the Victoria Hospital for Incurables is to be the form of the King Edward Memorial. A Committee has been appointed to confer with the Town Council on the subject. The cost of the extension is estimated at £30,000. A sum of £10,000 has already been raised.

**Chichester.** —At the annual meeting of the Governors of the Chichester Infirmary the plans for the renovation of the institution, which has been agreed upon as the best form for a memorial to the late King, were adopted. They provide for a new operating block and new wings, with accommodation for nurses and child patients. The Committee hopes to get the finances of the institution on a secure footing and then to ask the King to allow the infirmary to be styled the "Royal Infirmary."

**Mansfield.** —At the annual meeting of the Mansfield and Mansfield Woodhouse District Hospital it was proposed that the contemplated new extension of the hospital at an estimated cost of £6,000 should be made a memorial to the late King. The addition will provide for a further 31 beds which are much needed.

**Chiswick.** —It is proposed that the memorial should take the form of a cottage hospital. An anonymous donor has started a fund in the local *Times* for that object with the sum of £500.

**Loughborough** -Mr. W. B. Paget has offered a sum of money to build a new children's ward for the hospital as a memorial to the late King. The suggestion has been thankfully accepted, and a strong local committee has been formed to raise funds for the equipment and endowment of the ward.

**Sheffield.**—Several schemes are under consideration here, among others the establishment of a Children's Holiday Home. The proposal that a memorial fund should be raised to free all the medical charities of the city from debt and increase their efficiency is finding strong support. Both the Royal Infirmary and the Royal Hospital have recently been enlarged, and if the hospital memorial scheme is taken up, these two newly opened wings, cleared of debt, will be associated with the late King's name, while Jessop's and the Children's Hospitals will also be assisted.

**Woolwich.**—It has been decided to establish a hospital for Woolwich and Plumstead as a memorial to King Edward VII. A site in Nightingale Place has been suggested, and an appeal has been made to the Charity Commissioners to sanction a loan on the cottage hospital's reversion to the Monday bequest for the purpose of starting the new general hospital.

**Batley.** —At a recent special meeting convened to consider the various memorial proposals the following resolution was moved by the Mayor (Alderman J. Stubley, J.P.) and unanimously adopted :—"That the reign of his late Majesty King Edward VII. may be appropriately commemorated by the augmentation of the endowment fund of the Batley and District Hospital, and that to this end a subscription list be opened." A strong local committee has been formed and the appeal is being well responded to.

**Worthing.** —The Committee of the local hospital has considered the suggestion that the new extensions should be associated with King Edward's name as a memorial, but the suggestion has not been accepted.

**Tunbridge Wells.** -Active steps are now being taken to perpetuate the memory of his late Majesty King Edward VII. in the Borough. At a recent meeting of the Town Council the following resolution was proposed and carried unanimously :—"That in the opinion of this Council the memory of his late Majesty King Edward VII. can be best perpetuated in Tunbridge Wells by some additional endowment of the local hospital for the benefit of the sick poor, such endowments to be specifically designated by his late Majesty's name; and that his Worship the Mayor be requested to take such steps to give effect to this resolution as he may deem most desirable." The mover of this motion was Alderman Delves, J.P., and it is interesting to note, in passing, that his family has been on the Committee and taken a very active part in the management of the General Hospital since its inception in 1828. Alderman Delves is most constant in the performance of his duties at the hospital, in whose welfare he takes a keen interest. The above resolution is now being submitted to the inhabitants of the town and surrounding districts which the hospitals serve, and it is hoped that sufficient funds will be forthcoming to endow at least one bed in each hospital. The Electrical Department at the Tunbridge Wells General Hospital has been augmented by the presentation of a complete Finsen light apparatus; the apparatus is also adapted for charging accumulators, and was given by that generous supporter of the hospital, Sir David L. Salomons, Bart., who has now provided entirely free of cost to the committee a complete electrical department replete with the most recent and up-to-date apparatus. The inhabitants of the town and surrounding villages owe Sir David a deep debt of gratitude, his munificent gifts have proved of utmost value in hundreds of cases.

**Aberdeen.**—The memorial will take the form of a hospital for sick children, but a public statue is to be erected as well. An appeal fund has been opened and is being liberally subscribed to.

**Hastings.** —There is to be an extension of the local hospital, which stands in need of alteration and improvement. The standing committee has been increased and a special committee has been formed to deal with the whole matter and to collect funds for the proposed extension.

**Belfast.** —The Royal Victoria Hospital is to be improved and extended. The question of subsidiary memorials has also been considered, but no definite decision has as yet been arrived at.

**Poona, India.** —The Indian schemes are very various, and include statues and institutions. The hospitals, however, have been generously considered in India. Poona is to have a new hospital; so is Bassein. An influential committee has decided to erect a new hospital for women at Bannu. At Bombay the memorial is to take the form of a new hospital, a sanatorium, and a consumption home, together with a nursing institute, all of which establishments are urgently needed. At Calcutta a general memorial scheme has to be considered providing for hospital assistance and the erection of nursing homes and sanatoriums; no definite decision with regard to the form that the memorial is to take has yet been made. Lahore is to have a new medical college with an extension of the hospital. New hospitals have been decided on for Secunderbad, Kashmir, and the Central Provinces, while the MADRAS committee has selected as its memorial the establishment of a special hospital for consumptives. In many other places in India statues have been decided upon.

**The Dominions.**—None of the Dominions have as yet definitely decided as to the nature of the memorials they will select. At the Cape, in Canada, and in New Zealand the hospitals extension schemes have found favour, while in Australia there is a growing desire that the memorial should take a form which in some way expresses the late King's intimate association with sick charities and hospitals.

**Wimbledon.**—The hospital is to be rebuilt and extended as a memorial. Plans have been called for, and an estimate has been drawn up. To defray the expense a public subscription list has been opened.

**Warwickshire.**—This county has decided to extend the hospital and generally to promote its efficiency.

**Southampton.**—Two schemes have been put forward, one provides for the extension of the hospital, the other for the addition of a tower to St. Mary's Church. The former scheme appears to find the most favour locally and will doubtless be selected.

**Portsmouth.**—This town has decided upon the addition of a nursing home to the local hospital.

**Northumberland.**—The memorial will take the form of a new hospital.

**Norfolk.**—Here, too, an extension of hospital work has been decided upon. The interest that King Edward took in the hospital is well known, and it is felt that no more fitting memorial could be established to commemorate his reign than by the improvement and extension of the institution, the interests of which he had so much at heart.

**Shropshire.**—The memorial is to take the form of additions to the hospital and extension of philanthropic work.

**North Staffordshire.**—A new wing is to be built on to the infirmary, the accommodation of which is insufficient for the increase of patients. The scheme has received strong local support and the public is responding well to the appeal for funds to carry out the work.

**Birmingham.**—This city has already raised a sum of £27,000 for the erection of a new children's hospital and the erection of a statue, both to serve as a memorial to the late King.

**Bath.**—The committee here will have to consider two schemes, one providing for the establishment of a general hospital, the other for the erection of a statue of the late King.

**Bradford.**—The new infirmary will serve as a memorial to the late King. We have already given details of the scheme and the estimate of cost.

**Cumberland.**—A general hospital fund is to be established with the object of aiding the county hospitals and generally to increase their efficiency.

**Brighton, Chichester, Cambridge, Hastings, and Lancashire** have all decided to assist the local hospitals by improving and extending them. Local funds have been started with that object, and strong local committees have been formed.

**Leeds.**—The new infirmary scheme, to which attention has already been directed in these columns, is receiving adequate support.

**Devonshire.**—An attempt will be made to increase the general endowment of the Diamond Jubilee Hospital fund.

**Grimsby.**—It has been decided that the memorial is to take the form of a special convalescent fund.

**Haywards Heath.**—Here the memorial will be in the shape of a new cottage hospital.

**Ipswich** has decided upon a new sanatorium; the **Isle of Wight** upon extending the hospital and providing almshouses; **Oxford** upon a new sanatorium for consumptives; **Sittingbourne** upon a new cottage hospital; and **Worcestershire** upon a county sanatorium.

**Windsor.**—Here £20,000 is required to free the King Edward Hospital from debt and provide for its adequate endowment. It has been felt that this endowment fund will be the most suitable memorial to the late King.

**Melton Mowbray.**—A Committee has been formed to consider the possibility of erecting a Cottage Hospital at Melton Mowbray, as a memorial to King Edward VII.

**Leicester.**—The Executive Committee of the King Edward VII. Memorial at Leicester, appointed to consider the form the County Memorial should take, has resolved "That the County meeting be recommended to invest funds collected in the names of trustees chosen from the town and county of Leicester, and that the income of such funds be administered for the furtherance of the nursing of the sick of the town and county."

**Loughborough.**—As a memorial to King Edward VII., Mr. W. B. Paget, of Loughborough, has made a generous offer to the Governors of the Loughborough Hospital to buy additional land and build a new wing for children at the Hospital. A representative committee has been formed to collect subscriptions from the district for the provision of a fund to maintain the new wing. The Town Clerk (Mr. H. Perkins) has been elected Hon. Secretary and Mr. T. B. Jones Hon. Treasurer.

